# Supplementary material for: The Synergistic Effect of Thiamethoxam and Synapsin dsRNA Targets Neurotransmission to Induce Mortality in Aphis gossypii
Source: Int J Mol Sci. 2022 Aug 20;23(16):9388. doi: 10.3390/ijms23169388 (PMC9408958; doi:10.3390/ijms23169388)
Supplement: Supplementary file 1 [file ijms-23-09388-s001.zip › Qu-supplementary materials.pdf]

The synergistic effect of thiamethoxam and synapsin dsRNA targets neurotransmission to induce mortality in *Aphis gossypii*

Xueting Qu†, Sijia Wang†, Guangze Lin, Mingshan Li, Jie Shen and Dan Wang*

Department of Plant Biosecurity and MARA Key Laboratory of Surveillance and Management for Plant Quarantine Pests, College of Plant Protection, China Agricultural University, Beijing 100193, China

† These authors contributed equally to this work.

* Correspondence: dwang@cau.edu.cn

Supplementary Materials

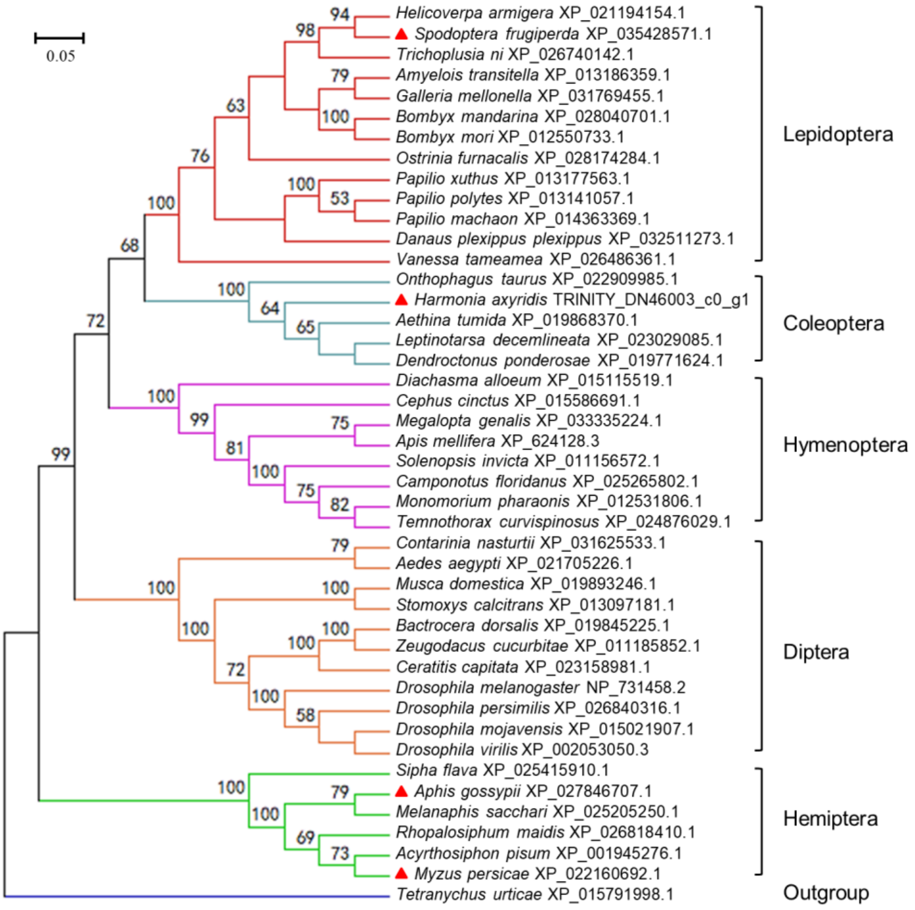

Figure S1. Phylogenetic analyses of *synapsin* in diverse insect species. Based on coding sequence alignment of synapsins from diverse insects, an unrooted NJ tree was generated. GenBank accession numbers and gene IDs from the transcriptome database were indicated.

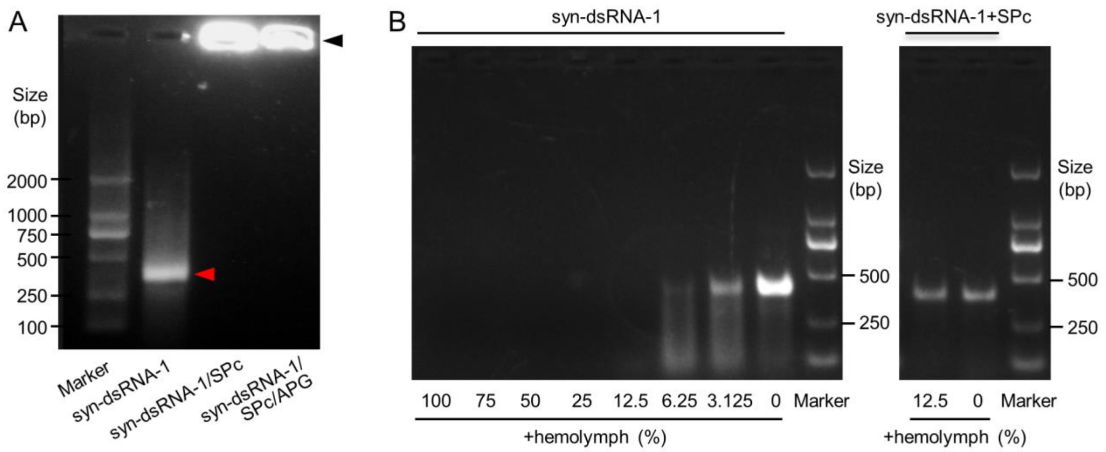

Figure S2. SPc protected syn-dsRNA from degradation. (A) The electrophoretic result showed the binding of syn-dsRNA to the nanocarrier SPc. The 313 bp syn-dsRNA-1 moved in the agarose gel (red arrowhead) during electrophoresis. After mixed with the nanocarrier SPc, dsRNA was stuck in the agarose gel (black arrowhead, lane 3) because of the neutralization of charges by SPc. The non-ionic surfactant APG did not affect the binding of syn-dsRNA-1 and SPc (black arrowhead, lane 4). (B) SPc protected dsRNA from degradation by aphid hemolymphs. Without SPc, syn-dsRNA was gradually degraded by high concentrations of aphid hemolymphs (left panel). In 12.5% and more hemolymphs, syn-dsRNA was completely degraded. By adding SPc (+SPc) with 1:1 mass ratio, syn-dsRNA was not, or mostly not degraded by 12.5% hemolymphs (right panel), indicating a protection of dsRNA by SPc.

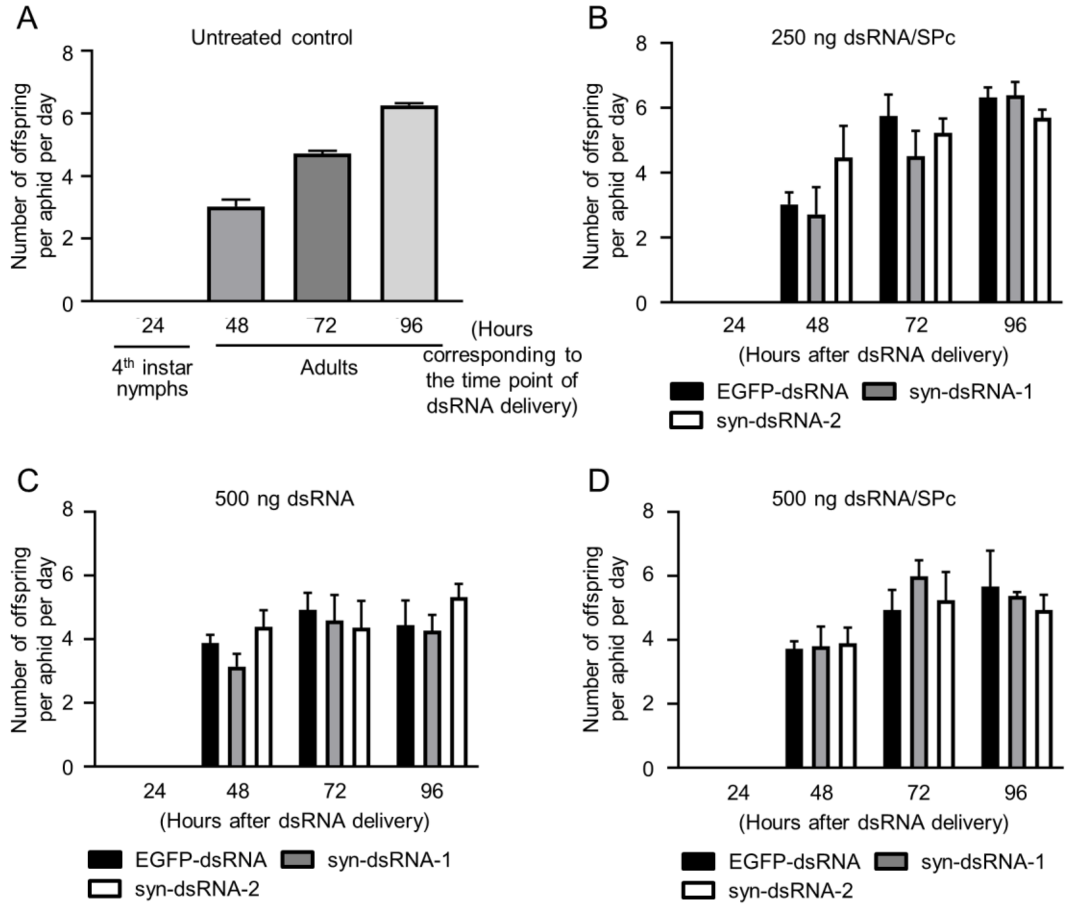

Figure S3. The offspring of survivors after syn-dsRNA delivery were comparable to that of untreated control and EGFP-dsRNA control. (A) Each adult aphid laid 3 to 6 nymphs per day. (B) The survivors treated by 250 ng syn-dsRNA showed normal number of offspring. (C, D) The survivors treated by 500 ng syn-dsRNA without (C) or with SPc (D) showed normal fertility.

Table S1. Volcano plot data for energy production related genes.

Table S2. Volcano plot data for neural function related genes.

Table S3. The primer sequences for qPCR.

**Gene**	**Primer name**	**Primer sequence (5'-3')**
beta−actin	act-F	GGGAGTCATGGTTGGTATGG
	act-R	TCCATATCGTCCCAGTTGGT
EGFP	EGFP-F	CCTAGTCCCCACAAACGAGT

	EGFP-R	CAAGCCGTCGAACATACTGG
synapsin	syn-F	GCAGGTTCAGGTATCACCCA
	syn-R	TAGATTCGCTTTCGCCGGTT
Epidermal growth factor receptor	EPS15-F	TGGTCGTATTTTGTTGCCGTC
substrate 15−like 1 (Eps15−like 1)	EPS15-R	AAGTAGCCATCCTGAACGC
Intersectin−1/ Dynamin associated	Dap160-F	GTCACATTACGACTTTGCCTTT
protein 160 (*Dap160*)	Dap160-R	R:CGGTGTTCTACCCCTGCTTA
Acetylcholinesterase	Ace-F	CTGCAGAAATGCACGTTGGT
	Ace-R	TCTTTCAAGGCTTGGGCGAT
Neuroligin−1	Nlg1-F	TCCGAAAGTATCCCGTGCTG
	Nlg1-R	GCGTGACGACAACCAATTCC
dynamin	dyn-F	ACCTGAGCAAGGACGACAAC
	dyn-R	TGTACACGCTGCGGTATTTG
stoned−B	stnB-F	GCCTTCACACGAAGCCAAAG
	stnB-R	CGCATTGGAGTAGGACTGGG
Synaptotagmin	Syt-F	AAGTCAATCTGGTGGTGACGG
	Syt-R	TGTCGGACCAGTGCCGTAAT
Endophilin−A	EndoA-F	ACGTCTGGAGCAAACCATCA
	EndoA-R	GTTGCGTGGTATTTGCCGT
octopamine receptor beta-2R	oct-F	TCAAGCCGCTCAAGTATCC
	oct-R	CCGTAGAAGATGGGCACGAA
Acetylcholine receptor subunit	AChRα-F	GACAGACACTGCACACGAGA
alpha−like 1 (*AChRα−like 1*)	AChRα-R	TTGTATGGTTCGTTCGGGCA
V−Type proton ATPase 21 kDa	V-Type-F	GGTTGGAGCCGCTTTAGGAA
proteolipid subunit	V-Type-R	GCTTCGCAGAAGATGACGGA

Table S4. The primer sequences for amplification of dsRNA.

**Gene**	**Primer name**	**Primer sequence (5'-3')**
EGFP	T7-EGFP-F	TAATACGACTCACTATAGGGCACAAGTTCAGCGTGTCCG
	T7-EGFP-R	TAATACGACTCACTATAGGGGTTCACCTTGATGCCGTTC
synapsin	T7-syn-1-F	TAATACGACTCACTATAGGGCGGCGATGTTGGAAGAAACC
	T7-syn-1-R	TAATACGACTCACTATAGGGCGTCTAGCCGGAATAGCTGG
synapsin	T7-syn-2-F	TAATACGACTCACTATAGGGGCAAACGGTTCTTTGGCGAT
	T7-syn-2-R	TAATACGACTCACTATAGGGCCCGACTTTGAGGACCACTG

Note: The underlined sequences were T7 promoter sequences.

Table S5. Toxicity of thiamethoxam to adults of *A. gossypii* at 48 h.

Insecticide	Regression equation (y=)	LC50 (mg/L)	95% confidence interval	Correlation coefficient
Thiamethoxam	0.439X+0.1655	5.249	3.771-7.296	0.9452

Video S1. Behavior of the EGFP-dsRNA treated melon aphid.

Video S2. Behavior of the syn-dsRNA treated melon aphid.
